# Interpretation of Root Canal Anatomy of Maxillary and Mandibular Permanent Canines in Saudi Subpopulation: A Cone-Beam Computed Tomography (CBCT) Study

**DOI:** 10.1155/2021/5574512

**Published:** 2021-02-12

**Authors:** Amal A. Almohaimede, Alanoud A. Alqahtani, Norah M. Alhatlani, Nouf S. Alsaloom, Shafia A. Alqahtani

**Affiliations:** Department of Restorative Dental Sciences, Endodontic division, College of Dentistry, King Saud University, P.O. Box 5967, Riyadh 11432, Saudi Arabia

## Abstract

The objective of this investigation was to evaluate the root canal anatomy of permanent canines in a Saudi subpopulation utilizing CBCT. A total of 1328 CBCT images of maxillary (634) and mandibular (694) permanent canines were collected from Saudi patients who visited the College of Dentistry at King Saud University in Riyadh. The images were analyzed for root and canal numbers, canal configuration types, and symmetry between the bilateral canine teeth in each arch. Gender, age, and tooth position were recorded. The Kappa test was used for both inter- and intraexaminer reliabilities. Data were analyzed with the chi-square test at a *P* value ≤ 0.05. There were no two-rooted teeth in maxillary canines, and 1.89% had two canals. Double roots and double canals in mandibular canines were found in 2.88% and 9.94% of the teeth, respectively. Type I canal configuration was more common in maxillary canines (97.94%) compared to mandibular canines (92.07%). Maxillary canines showed higher bilateral symmetries of root and canal numbers and canal configuration types (100%, 98.1%, and 97.9%, resp.) compared to mandibular canines (97.1%, 90.1%, and 92.1%, respectively). The majority of maxillary and mandibular canines had a single root with a single canal and type I canal configuration. Mandibular canines are characterized by having more than one root and canal and alterations in root canal configurations compared to maxillary canines.

## 1. Introduction

Awareness of the canal anatomy and its alterations, thorough debridement and shaping of all pulp canals, and complete canal filling are essential steps for favorable root canal treatment [1]. Missing the canal and leaving it without treatment may negatively affect the treatment outcome [2].

Researchers have used various methods and techniques to assess root canal morphology, including serial sectioning [3, 4], canal staining and clearing [5, 6], conventional and digital radiographic examination [7], and microcomputed tomography (*µ*CT) [8]. In the late 1990s, three-dimensional scans of the maxillofacial skeleton were developed and since that time, it has become available for dental offices [9]. This method is characterized by a lower radiation dose and shorter acquisition time than conventional computed tomography (CT) [10]. Moreover, cone-beam computed tomography (CBCT) is an accurate and noninvasive method that provides a presentation of the root canal system in three dimensions [11], and its reconstructions showed a very strong correlation with the histologic sections [12].

Several investigators have investigated different variations in root canal morphology [4, 6, 13]. Vertucci classified the root canal morphology into eight types, and many researchers have used it for root canal system classification [6].

Several studies have demonstrated that root canal morphology varies according to race, ethnic group [14–16], and gender [17, 18]. Therefore, these variations should be acknowledged in the pretreatment analysis for root canal therapy.

Mandibular and maxillary permanent canines mostly have one root with one canal [13, 19]. However, several studies have reported different anatomical alterations of maxillary and mandibular permanent canines of different races and genders [20–22].

The objective of this investigation was to evaluate the root canal anatomy of permanent maxillary and mandibular canines in a Saudi subpopulation utilizing CBCT.

## 2. Materials and Methods

The ethics committee at King Saud University, College of Medicine (IRB Project No. E-17-2742), approved this study. A total of 1328 CBCT images of maxillary and mandibular permanent canines were obtained from Saudi patients (565 males and 763 females) aged between 18 and 74 years. These patients attended the Radiology Department at the College of Dentistry at King Saud University in Riyadh between the years 2015 and 2019.

The samples were selected based on the availability of CBCT images of maxillary and/or mandibular canines with full root formation. Excluded images were low-quality CBCT images, previously root canal treated or initiated teeth, presence of root resorption or periapical lesions, presence of coronal or postrestorations, and teeth with immature apices. The total final sample consisting of 634 maxillary canines and 694 mandibular canines was observed and analyzed for root and canal numbers and the configuration types of root canals based on Vertucci's classification [6]. Moreover, the symmetry of roots and canals and the canal configuration between the bilateral canine teeth in each arch were observed and analyzed. Gender, age, and tooth position were reported.

The CBCT images were analyzed at the Radiology Department of the College of Dentistry at King Saud University, by one endodontist and three trained interns for tooth position, root and canal numbers, and canal configuration types. A professional oral radiologist was consulted. The Planmeca Romexis Viewer software was used for image assessment (Planmeca, Roselle IL).

Radiological images were obtained by a professional technician according to the manufacturer's recommended protocol using different CBCT machines: Planmeca ProMax 3D (PLANMECA, Roselle, IL, USA) and CS9300 3D digital imaging system (Carestream, Rochester, NY). The exposure time was 3–15 seconds. The slice thickness was 0.2 mm thick viewed from the coronal to apical region, and the voxel size was 75–600 *μ*m, with small or large fields of view (FOVs) included.

Twenty CBCT images (with 10 maxillary and 10 mandibular canines) were randomly selected according to the evaluation criteria to measure the intra- and interexaminer reliabilities. Images were identified for tooth position, root and canal numbers, and root canal configuration types. For intraexaminer agreement, the same images were reassessed by the same examiner after one week. The interexaminer agreement was measured among the four different examiners.

### 2.1. Statistical Analysis

For both inter- and intraexaminer reliabilities, the Kappa test was used [23]. For data analysis, chi-square test was used using SPSS 22 software (SPSS Inc, Chicago, IL), and *P* value ≤ 0.05 indicated statistical significance.

## 3. Results

For interexaminer reliability, kappa test values were 1 (almost perfect agreement) for the number of roots, 1 (almost perfect agreement) for the number of canals per canal, and 0.8 (substantial agreement) for the configuration of root canals. For intraexaminer reliability, kappa test values were 1 for all examiners regarding root and canal numbers. For root canal configuration types, kappa test values were 1 for the first and fourth examiners, 0.85 for the second examiner, and 0.95 for the third examiner. Kappa test values verified the reliability of the measurements conducted by the four examiners.

The frequency of teeth according to gender and tooth position is summarized in Table 1.

**Figure 1:**
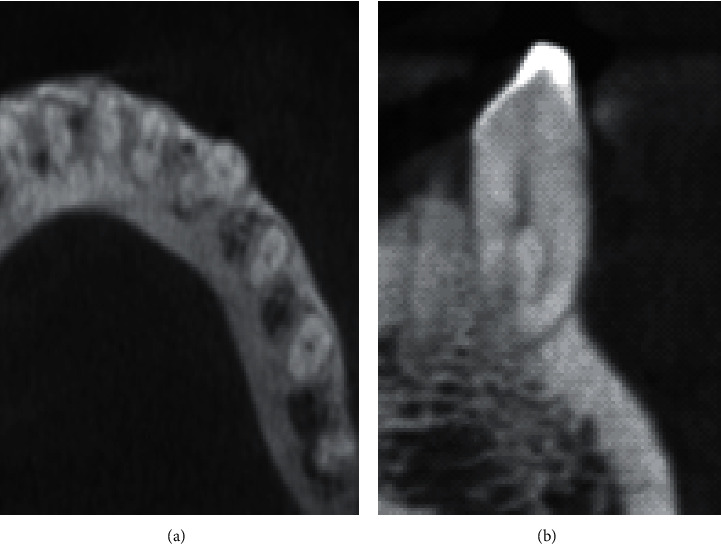
Cone-beam computed tomography image for mandibular canine with two roots. (a) The axial plane. (b) The coronal plane.

The number of roots recorded was up to two roots in 20 canines (1.5%) **(**Figure 1**)**, and the majority were one-rooted teeth in 1308 canines (98.5%). The females harbored a greater number of two roots (14 canines/1.8%) than males (6 canines/1.1%). However, there was no statistically significant difference noted (*P* = 0.362). A significant difference was noted between the number of roots and the tooth position (*P* = 0.000). Of the 694 lower canines, 674 (97.11%) teeth were single-rooted and 20 (2.88%) teeth were double-rooted. However, all the 634 (100%) maxillary canines had one root.

The number of canines recorded with two canals was 81 (6.1%), and the majority of teeth had one canal (1247 canines, 93.9%). Females harbored a larger number of canines with two canals (55/7.2%) than males (26/4.6%). However, no statistically significant difference was noted (*P*=0.063). A significant difference was noted between the number of canals and the tooth position (*P*=0.000). Of the 694 mandibular canines, 625 (90.05%) had a single canal, and 69 (9.94%) had double canals. Of the 634 maxillary canines, 622 (98.1%) had a single canal, and 12 (1.89%) had double canals.

Type I canal configuration was mostly observed in 1260 canines (94.9%), with a statistically significant difference between the other configuration types (*P*=0.005), followed by Type V in 24 canines (1.8%), Type III in 23 canines (1.7%), Type II in 14 teeth (1.1%), Type IV in 5 canines (0.4%), and Type VII in two canines (0.2%). Regarding gender, Types I, II, III, V, and VII were common in females, whereas Type IV was more frequent in males (*P*=0.005) (Table 2).

A significant relationship was noted between canal configuration and the tooth position (*P*=0.003). The mandibular canines showed more variations in canal configuration than the maxillary canines. The Type I configuration was more common in maxillary canines (97.94%) compared to mandibular canines (92.07%). However, Types II, III, VI, V, and VII were more common in mandibular canines compared to maxillary canines (Table 3).

Within the same maxillary arch, both left and right canines existed in 313 patients. In total, 100% of the teeth showed symmetrical root numbers, 98.1% showed symmetrical canal numbers, and 97.9% showed symmetrical canal configurations. However, in the mandibular arch, both left and right canines existed in 347 patients. In total, 97.1% of the teeth demonstrated a symmetrical number of roots, 90.1% showed a symmetrical number of canals, and 92.1% showed a symmetrical canal configuration.

Regarding age, no difference was observed between the different age groups and the number of roots (*P*=0.923). However, a statistically significant difference was noted between the different age groups and the number of canals and the type of canal configuration (*P*=0.023 and *P*=0.000, respectively) and the older age group (>65 years old) showed more complicated root canal anatomy. The results are summarized in Tables 4 and 5.

## 4. Discussion

This study showed anatomical alterations in the morphology of the root canal system of human permanent canines in both arches in a Saudi subpopulation. Dentists must be knowledgeable of the anatomical alterations in the root canal system to avoid iatrogenic procedural errors that arise from inadequate knowledge. Therefore, since 1870, the literature has documented studies on the anatomy of the root canal system of teeth in different populations using different and improved analysis techniques [24, 25]. In this study, the CBCT technique was used to provide a third dimension to analyze the root canal anatomy. The efficiency of CBCT in revealing the morphology of the root canal has been studied and compared with the standard methods in evaluating root canal morphology. Kajan et al. found that CBCT and clearing and staining methods were comparable in detecting the number of root canals of teeth in both arches [26]. Another study showed that CBCT is better than the clearing technique in detecting Type I Vertucci classification [27]. The technique selected to evaluate the anatomy of the root canal should be valid, simple, noninvasive, feasible, and reproducible [28, 29].

The present study investigated the maxillary and mandibular permanent canines' root canal morphologies in a Saudi subpopulation. The incidence of double roots and canals in lower canines in this study was 2.88% and 9.94%, respectively. Our results are in accordance with the findings of Mashyakhy in his study in a Saudi Arabian population, where he found that 2.7% of the lower canines had double roots and 9.3% had double canals [30]. However, the finding of double roots and canals in mandibular canines was less than that reported in a previous study in a Saudi population at 0.2% and 4.6%, respectively [31]. This difference could be attributed to their smaller sample size. Comparable results to our findings were found in the Turkish population regarding the presence of two-rooted mandibular canines (3.1%) [32] and the Syrian population (2.15%) [33]. In Iranian populations, Aminsobhani et al. and Rahimi et al. found higher levels than that noted in our study in mandibular canines with two roots at 4.7% and 12.08%, respectively [34, 35]. However, our rates are greater than those of Pecora et al. in Brazil (1.7%) [36], Zhao et al. (0.7%) in the Chinese population [37], Singh and Pawar in the South Asian Indian population (0%) [38], Zhengyan et al. (0.8%) in the Chongqing population [39], Pan et al. (1.21%) in the Malaysian population [40], and Soleymani et al. (1.3%) in the Iranian population [22]. The presence of two canals in lower canines in other different populations ranged between 0 and 15.1% [22, 32, 34, 36, 37]. These morphological variations could be attributed to different ethnic groups.

No two-rooted teeth in maxillary canines were noted in the present study. In total, 98.1% had one canal, and only 1.89% had two canals. Our results are consistent with the findings of Mashyakhy in a Saudi population (100% with one root; 99% with one canal, and 1% with two canals) [30]. Additionally, the findings are consistent with those reported in Malaysian and Iranian populations (100% had one root and one canal in the upper canines in both populations) [40, 41]. Most of the recorded literature about the presence of two roots in maxillary canines involved case reports [42–44]. This finding indicates that the existence of two roots or canals in maxillary canines is infrequent.

This study showed that Type I canal configuration was found in most mandibular canines (92.07%), followed by Type III (2.88%) and Type V (2.44%). Our results agree with those reported in a previous investigation in a Saudi subpopulation, where most canal configurations were Type I (90.7%) followed by Type III (6.1%) and Type V (3.2%) [29]. However, another study in a Saudi subpopulation found that most mandibular canines had a Type I canal configuration (95.4%) followed by Type II (2.6%) and Type III (1.8%) [31]. These differences could be explained by the smaller sample size compared to our study. These findings are similar to those reported in several studies in different populations, in which the Type I canal configuration had the highest incidence among other configuration types [22, 33–40] in lower canines.

Concerning maxillary canines, this study showed that the Type I canal configuration was mostly observed (97.94%) followed by Type V (1.1%) and Types II and III (0.47%). Our results are slightly different from what Mashyakhy found in his study in a Saudi Arabian population, where he documented Type I (99%) and Type III (1%) canal configurations in maxillary canines [30]. This finding could be explained by the difference in the sample size between both studies. Comparable results were also found among the Turkish population in a study using the clearing technique in maxillary canines, where the Type I canal configuration was mostly observed (93.48%) followed by Type III (4.35%) and Type V (2.17%) [23]. In contrast, a Type I canal configuration was found in all examined maxillary canines (100%) in a Malaysian population [40].

Regarding gender, the present study reported no differences in root and canals numbers between genders. However, a significant difference was noted between genders regarding canal configuration (*P*=0.005). Types I, II, III, V, and VII were more frequent in females than in males, whereas Type IV was more frequent in males than in females. These results could be explained by the larger sample size in females (763) than in males (565). Our findings are in partial agreement with those reported by Mashyakhy in a Saudi Arabian population, where no significance in the root and canal numbers was noted between genders in canines in both arches. Nonetheless, a significant difference was noted between genders regarding canal configurations. Types III and V were more common in females than males, and Type I was more common in males than females [30]. Similarly, in another study in a Saudi population on lower canines, no significant difference was found between gender and the number of roots. However, there was a notable difference between root canal configuration and gender. Types II, III, and IV canal configurations were more frequent in males and Type I was more frequent in females [31]. In a Malaysian subpopulation, similar results were reported regarding the absence of differences between genders in the number of roots and canals [40]. In contrast to our results, in an Iranian population, males have a higher incidence of double roots and canals in lower canines [22]. On the other hand, Martins et al. reported that females had more root numbers than males in lower canines in a Portuguese population [18].

The current study examined bilateral symmetry in canines in both arches. In lower canines, our results showed high bilateral symmetry for the root numbers (97.1%), number of canals (90.1%), and canal configuration (92.1%). These results agree with the findings of Mashyakhy (95.5% for root numbers, 91.1% for canal numbers, and 90.1% for canal configuration) [30] and Al-Dahman et al. (97.7% for the root numbers and canal configuration) [31] in a Saudi Arabian population. Our results are also consistent with that found in an Iranian population, where Soleymani et al. reported 95.4% bilateral symmetry in root numbers and canal configuration in lower canines [22]. In contrast, Kayaoglu et al. reported 28% bilateral symmetry in canal numbers in mandibular canines in a Turkish population [32].

Our results in maxillary canines showed higher bilateral symmetry than mandibular canines for roots (100%), canals (98.1%), and canal configuration (97.9%). This finding was consistent with Mashyakhy's study in a Saudi population (100% for root numbers, 98.9% for canal numbers, and 98.9% for canal configuration) [30]. Karataslioglu and Kalabalik reported in their study in a Turkish population that 96.6% of the maxillary canines had bilateral symmetry in the root canal configuration [45].

The effect of age on tooth anatomy has rarely been studied. In our study, the results showed that the older age group (>65 years old) had a higher frequency of two canals than the younger age groups and a more complex root canal configuration. These results can be clarified by the physiological change in root canal system morphology during aging through the formation of secondary dentine. Similar results were found by Karataslioglu and Kalabalik in a Turkish population [45] and by Martins et al. in a study on the effect of age on the root canal system configuration using CBCT [46]. In contrast, previous studies in Turkish and Chongqing populations showed that younger patients had more frequent multiple canals detected than older patients [32, 39].

The data of the current study were limited to one place, where the images were analyzed from one center in one region with variations in the number of samples among the different age groups and gender. Therefore, future studies are recommended to include different regions with different centers with increasing the sample sizes.

## 5. Conclusion

Within the limitations of this study in a Saudi subpopulation, the majority of canines in both arches were single-rooted with single canal and Type I canal configuration. Lower canines demonstrated a higher incidence of double roots and canals than upper canines with more variations in canal configurations. The older age group (>65 years old) showed more complex root canal anatomy. Lower canines showed higher asymmetries in the number of roots and canals and in the type of canal configuration compared to upper canines. Therefore, clinicians need to consider these morphological variations in the pretreatment analysis for the root canal therapy.

## Figures and Tables

**Table 1:** The frequency of teeth according to gender and tooth position.

	Frequency of teeth (%)
*Gender*	
Male	565 (42.5%)
Female	763 (57.5%)

*Tooth position*	
Maxillary right canine	321 (24.2%)
Maxillary left canine	313 (23.6%)
Mandibular left canine	347 (26.1%)
Mandibular right canine	347 (26.1%)

**Table 2:** Variations in canal configuration according to gender.

	Frequency of teeth (%)	
	Male (565)	Female (763)
*Canal configuration type*		
Type I	542 (95.9%)	718 (94.1%)
Type II	1 (0.2%)	13 (1.7%)
Type III	7 (1.2%)	16 (2.1%)
Type IV	5 (0.9%)	0
Type V	10 (1.8%)	14 (1.8%)
Type VI	0	0
Type VII	0	2 (0.3%)
Type VIII	0	0

**Table 3:** Variations in canal configuration according to tooth position.

	Frequency of teeth (%)	
	Maxillary canines (634)	Mandibular canines (694)
*Canal configuration type*		
Type I	621 (97.94%)	639 (92.07%)
Type II	3 (0.47%)	11 (1.58%)
Type III	3 (0.47%)	20 (2.88%)
Type IV	0	5 (0.72%)
Type V	7 (1.1%)	17 (2.44%)
Type VI	0	0
Type VII	0	2 (0.28%)
Type VIII	0	0

**Table 4:** Prevalence of root and canal number among different age groups.

Age range groups (years old)	Prevalence of teeth		Prevalence of teeth	
	With one root (%)	With two roots (%)	With one canal (%)	With two canals (%)
18–23	98	2	94.1	5.9
24–29	99.2	0.8	93.8	6.2
30–35	98.6	1.4	96.4	3.6
36–41	97.6	2.4	88.1	11.9
42–47	98.8	1.2	97.6	2.4
48–53	98.1	1.9	91.7	8.3
54–59	98.7	1.3	98.7	1.3
60–65	96.9	3.1	92.3	7.7
>65	100	0	85.7*∗*	14.3*∗*

*∗*Significant at *P* ≤ 0.05.

**Table 5:** Prevalence of different types of canal configurations among different age groups.

Age range groups (years old)	Canal configurations					
	Type I	Type II	Type III	Type IV	Type V	Type VII
18–23	94.9%	0.4%	0.8%	1.2%	2.8%	0
24–29	94.6%	1.2%	2.5%	0	1.7%	0
30–35	97.1%	0	2.1%	0	0.7%	0
36–41	89.7%	0.8%	4.8%	0	4.8%	0
42–47	98.8%	1.2%	0	0	0	0
48–53	91.7%	1.9%	1.9%	0	4.6%	0
54–59	100%	0	0	0	0	0
60–65	95.4%	0	0	1.5%	0	3.1%
> 65	85.7%*∗*	3.6%*∗*	7.1%*∗*	0	3.6%	0

*∗*Significant at *P* ≤ 0.05.

## Data Availability

The data used to support the findings of this study are available from the corresponding author upon request.
